# Antenatal jaundice instruction and acute bilirubin encephalopathy in Nigeria

**DOI:** 10.1038/s41390-023-02887-6

**Published:** 2023-12-02

**Authors:** Richard P. Wennberg, Zainab O. Imam, David D. Shwe, Laila Hassan, Zubaida L. Farouk, Lindsey E. Turner, Ann M. Brearley, Tina M. Slusher, Stephen Oguche

**Affiliations:** 1 Emeritus, Department of Pediatrics, University of California, Davis, Davis, CA USA; 2 Department of Pediatrics, Lagos State University Teaching Hospital, Lagos, Nigeria; 3 Department of Pediatrics, University of Jos, Jos, Nigeria; 4 Department of Pediatrics, Ahmadu Bello University, Zaria, Nigeria; 5 Department of Pediatrics, Bayero University, Kano, Nigeria; 6 Division of Biostatistics, School of Public Health, University of Minnesota, Minneapolis, MN USA; 7 Biostatistical Design and Analysis Center, Clinical and Translational Science Institute, University of Minnesota, Minneapolis, MN USA; 8 Department of Pediatrics, University of Minnesota, Minneapolis, MN USA; 9 Department of Pediatrics, Hennepin Healthcare, Minneapolis, MN USA

## Abstract

**Background:**

Acute Bilirubin Encephalopathy (ABE) is common in Nigeria. Parents’ inability to recognize jaundice and delays in seeking care are significant barriers to its prevention.

**Methods:**

We compared associations of (1) interactive antenatal maternal jaundice instruction with postnatal reinforcement, (2) standard postnatal instruction, and (3) no maternal instruction with the incidence of ABE among 647 jaundice admissions stratified for risk factors identified in initial descriptive analysis.

**Results:**

Eighty-three (83/647;12.8%) admissions developed ABE including eleven jaundice-related deaths. ABE was present at admission in 20/22 (90.9%) if mothers received no jaundice instruction and no antenatal care, 42/182 (23.1%) if received antenatal care but no instruction, 16/95 (16.8%) if received postnatal instruction only, and 4/337 (1.2%) if mothers received both antenatal and postnatal instruction (*p* < .001). ABE was highly associated with out-of-hospital delivery, number of antenatal clinic visits, and birth attendant, but these risks were mitigated by antenatal/postnatal instruction. Admission rates with bilirubin levels below treatment guidelines (12 mg/dL) were higher following instruction (30.7%) than with no instruction (14.4%). Limiting subjects to those meeting admission criteria increased ABE rates in all groups without altering conclusions.

**Conclusion:**

Interactive antenatal instruction with postnatal reinforcement resulted in timely care seeking and a lower incidence of ABE.

**Impact:**

- Empowering mothers to participate in neonatal jaundice management is critical in low-income countries where jaundice monitoring and follow up are unreliable.
- Instructing mothers about jaundice in antenatal clinics with postnatal reinforcement is more effective than standard postpartum instruction in facilitating jaundice detection, timely care seeking, and lowering the incidence of acute bilirubin encephalopathy (ABE).
- Antenatal training also mitigates risks for ABE associated with out-of-hospital deliveries, limited antenatal care, and unskilled birth attendants.
- Impact: Adding structured jaundice instruction in antenatal clinics could greatly reduce bilirubin induced brain injury in countries where ABE is common.

## Introduction

In high-income countries (HIC), a systems approach to monitor neonatal jaundice/hyperbilirubinemia involves pre-discharge jaundice assessment, pre-discharge parent instruction, and follow up guided by the newborn’s clinical status and risk of developing significant hyperbilirubinemia based on an hour-specific nomogram.^1,2^ The system generally works well in HIC countries^3–5^ resulting in a very low incidence of acute bilirubin encephalopathy (ABE) and residual kernicterus spectrum disorder (KSD),^6^ but less well in low-middle-income countries (LMICs) where bilirubin monitoring is rare and follow-up care is inconsistent.^7,8^

ABE is common and a frequent cause of neonatal death^9–14^ in regions where glucose-6-phosphate dehydrogenase deficiency (G6PDd)^14–17^ and out-of-hospital births^7,12,16,18^ are prevalent. Timely care seeking is critical since most neonates with ABE are already symptomatic when admitted to institutions capable of treating severe hyperbilirubinemia.^19–21^ In communities lacking close monitoring by health workers, identifying jaundice and seeking timely care to prevent ABE is often up to the parents.^22–24^

Several studies link delayed treatment and ABE to low socio-economic markers such as home births, lack of antenatal care, untrained birth attendants, financial status, and family hierarchies that restrict timely medical care.^8,16,25,26^ Others have focused on parents’ lack of knowledge and attitude about jaundice and/or potential for complications.^27–29^ In either case, the resulting failure to recognize jaundice or ineffective actions such as trials of traditional medicines, antibiotics, or unfiltered (often early morning) sun exposure only delay proper therapy.^12,20,29–32^

We previously documented a high rate of out-of-hospital births in the Nigerian population admitted for jaundice treatment making traditional postpartum instruction about jaundice problematic.^20^ Ninety-five percent of mothers had received antenatal care for at least two visits while only 63% of mothers gave birth in hospital settings.^24^ By introducing interactive group instruction about jaundice and its risks in antenatal clinics with reinforcement following delivery we reached a larger population of parents in a less stressed environment than instruction provided only following birth.^23^

In this study, we analyze a subset of the original data files^23^ to identify (1) associations of parents’ antenatal care choices and postnatal newborn care decisions with the occurrence of ABE and (2) determine whether identified high risk choices can be mitigated by providing mothers information about jaundice and its risks before birth with reinforcement after delivery.

## Methods

### Study sites

This multisite cross-sectional observational study was conducted in four centers in Nigeria: Massey Children’s Hospital in Lagos, and hospitals affiliated with university teaching programs in Jos, Zaria and Kano. The protocol was reviewed and approved by Institutional Review Boards of each participating hospital. Mothers’ verbal consent was obtained during the admission interview.

### Training protocols

Training curricula appropriate for physicians, midwives, nurses, and community health extension workers (CHEWs), and expectant mothers were developed, and peer reviewed by members of the Stop Kernicterus in Nigeria (SKIN) consortium who were practicing pediatricians.

Group training of healthcare providers was conducted over an 18-month period prior to introducing instruction to mothers. Maternal instruction was offered in selected antenatal clinics from June 2015 through November 2015. Antenatal training was conducted by physicians following SKIN protocols and consisted of group interactive sessions lasting 20–40 min in participating antenatal clinics. Postnatal instruction was less structured and usually reinforced with written information. It was offered in postpartum wards, usually one-on-one or, in the case of early discharge or out-of-hospital delivery, at a follow up clinic 2–4 days after birth.

### Subject selection

During the six months training period 659 neonates >24 h of age were admitted for neonatal jaundice/hyperbilirubinemia and phototherapy in the four participating centers. Following parent interviews, study participants were divided into three groups: (1) mothers had participated in an antenatal instruction session with post-delivery reinforcement, (2) mothers received instruction about jaundice but only following birth, (3) mothers did not receive antenatal or postnatal instruction (not all clinics offered the program). A limited number of infants (*N* = 12) whose mothers received antenatal, but no postnatal instruction was excluded from analysis leaving 647 cases.

### Jaundice management

The bilirubin threshold criterion for phototherapy in term infants at participating centers is 12 mg/dL, lower than NICE and AAP standards. The guideline recognizes uneven quality of diagnostic, treatment, and transport services in LMICs. Total serum bilirubin (TSB) and newborn blood types were rarely determined before discharge. G6PD levels were not measured, and, while maternal and newborn blood types and Rh status were documented in most cases following admission for phototherapy, direct antibody testing was uncommon. Phototherapy units had minimal irradiance levels of 15 mW/cm^2^/nm. ABE was classified as mild, if patients received a BIND score of 1–3 on a 9-point evaluation^33^ or 1–4 using a 12-point system.^34^

### Maternal instruction content

**Fig. 1:**
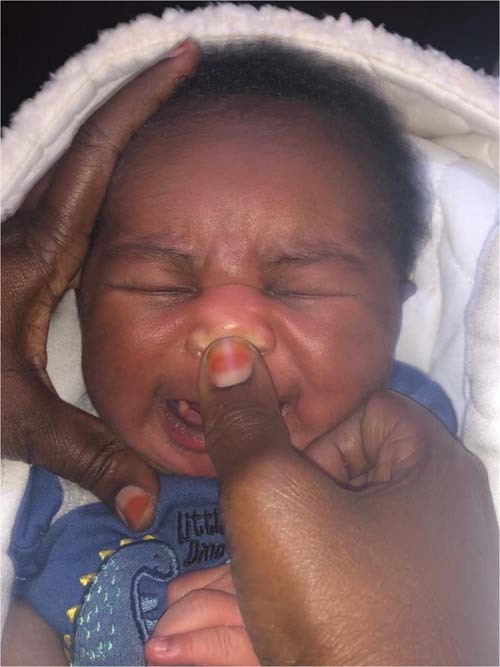
The Blanch Test. To identify jaundice in pigmented skin, press the top of the nose with finger or thumb for 3–5 s and note the skin color as pressure is released.

Subject matter of interactive group sessions included an overview of neonatal jaundice—jaundice is common but may be severe with hemolytic conditions and produce ABE and KSD if not treated promptly—followed by instructions on what parents can do to prevent ABE. Early recognition of jaundice and evaluation by clinic or hospital personnel were emphasized. Mothers were shown how to recognize jaundice by nose compression (“blanch test,” Fig. 1), facilitated in some centers by a disposable color reference card calibrated for dark skin to reflect TSB levels of 5 and 10 mg/dL^35^ (BiliStrip, Bilimetrixusa.org). They were taught to seek care immediately if jaundice is detected by the blanch test or yellow conjunctivae, to avoid ineffective therapies for jaundice, the importance of breast feeding, and the early signs of ABE. Since G6PDd is the most common hematologic cause of severe jaundice and ABE in Nigeria, yet not routinely screened for, instruction assumed all neonates were deficient and mothers were taught to avoid a list of hemolytic triggers. Updated training materials used in this study can be found on the Stop Kernicterus International website (skibilimetrixusa.azurewebsites.net) presented in multiple languages and supplemented by both slide and video presentations.

In addition to interactive instruction, posters were displayed in clinics depicting jaundice detection/action and avoidance of hemolytic agents (e.g., moth balls, camphor). In Kano, a radio jingle conversation between a health worker and mother described how to identify jaundice, its potential complications, and the importance of seeking immediate medical attention at a hospital or clinic ran twice daily for 4 months. Potential confounding contributions of posters and jingles to parent decisions were not evaluated.

### Statistical analysis

Descriptive analyses focused on care decisions by parents and other demographic and clinical variables, and their associations with risk for ABE. Documented parent care choices included frequency of antenatal clinic visits, birth site selection (hospital, clinic or home/other), and birth attendant selection (physician, midwife, unskilled, or other).

Normally distributed continuous variables were summarized using mean (standard deviation) values, and associations with ABE status or type of instruction were assessed using *t* tests. For variables not normally distributed, median (interquartile range) values and Kruskal-Wallis rank-sum tests were applied (e.g., age at admission, gravida, and admission TSB). Categorical variables were summarized using number and percent occurrence and analyzed using chi-squared tests.

The association of maternal training with ABE occurrence, both independently and as a mitigator of identified risks involving parent care decisions, was examined using chi-squared tests and multiple logistic regression. Multiple logistic regression models were used to estimate the effect of maternal instruction on the odds of ABE, including both unadjusted models and models adjusted for number of prenatal visits, birth site, birth attendant, and center. Global associations between categorical variables and ABE occurrence were assessed using Type III ANOVA tests (i.e., to assess whether the odds of ABE varied significantly across all types of maternal instruction). Pairwise associations between individual levels of a categorical variable and ABE occurrence were assessed using Wald *t* tests (e.g., to assess whether the odds of ABE differed between postnatal instruction and no instruction). All analyses were conducted using R statistical software, version 4.1.2, running in RStudio, version 2022.07.2.

## Results

### Study population

Demographics, socio-economic characteristics, birth situations, and laboratory data on 647 admitted neonates are summarized in Table 1 including their association with ABE. Every characteristic listed, except for sex and gravida, is strongly associated with the presence of ABE in this population. The neonates with ABE tended to be older, weigh less, have higher admission TSB levels, and have risk factors for hemolysis. Their mothers tended to be younger, had received fewer antenatal visits, were more likely to have had birth outside a hospital, and had a birth attendant other than a physician or midwife.

**Table 1:** Descriptive statistics and associations with ABE.

	No ABE	ABE
*N*	564	83
Age at Admission, days	5 [3, 7]	7 [5, 9] *
Admission Weight, grams	2924.62 (644.25)	2662.44 (505.20) *
Sex		**
Male	339 (60.1)	57 (68.7)
Female	216 (38.3)	26 (31.3)
Unknown	9 (1.6)	0 (0.0)
Mother’s Age, years	28.55 (6.18)	26.37 (5.55) ***
Gravida	2 [1, 4]	2 [1, 4] ****
Number of Prenatal Visits		*
0	2 (0.4)	22 (26.5)
1 to 3	91 (16.1)	10 (12.0)
4 to 5	248 (44.0)	27 (32.5)
6 or more	188 (33.3)	23 (27.7)
Unknown	35 (6.2)	1 (1.2)
Birth Site		*
Hospital	381 (67.6)	23 (27.7)
Clinic	76 (13.5)	20 (24.1)
Home/Other	106 (18.8)	40 (48.2)
Unknown	1 (0.2)	0 (0.0)
Birth Attendant		*
Physician/Midwife	423 (75.0)	26 (31.3)
CHEW^a^	113 (20.0)	28 (33.7)
TBA^b^/Other	27 (4.8)	28 (33.7)
ABE Status		*
No ABE	564 (100.0)	0 (0.0)
Mild ABE	0 (0.0)	28 (33.7)
Moderate/Severe ABE	0 (0.0)	44 (53.0)
Death	0 (0.0)	11 (13.3)
Center		*
Jos	206 (36.5)	4 (4.8)
Kano	234 (41.5)	31 (37.3)
Lagos	60 (10.6)	41 (49.4)
Zaria	64 (11.3)	7 (8.4)
Laboratory Data		
Admission TSB mg/dL	12.50 [8.80, 16.80]	29.80 [22.95, 33.55]*
Potential Hemolysis		*
No incompatibility	334 (59.2)	26 (31.3)
ABO incompatibility	103 (18.3)	22 (26.5)
Rhesus incompatibility	20 (3.5)	6 (7.2)
Unexplained Hct ≤35	4 (0.7)	18 (21.7)
Suspected sepsis	0 (0.0)	10 (12.0)
Missing Data	101 (17.9)	1 (1.2)

Continuous variables were summarized using mean (standard deviation) except for age at admission, gravida and admission TSB, which were summarized using median [interquartile range]. Categorical variables were summarized using number (percent). *P*-values are from *t*-tests for all continuous variables except age at admission, gravida and admission TSB, for which Kruskal-Wallis rank-sum tests were used, and from chi-squared tests for all categorical variables.

*P* Values: *p < 0.001, **0.209, ** 0.003, ****0.281.

^a^CHEW Community Health Worker.

^b^TBA Traditional Birth Attendant.

^c^Hct hematocrit.

### Associations with type of maternal instruction

Demographics, socio-economic characteristics, birth situations, and laboratory data on the 647 admitted infants are summarized again in Table 2, this time with the association of each characteristic with type of maternal jaundice instruction. As before, every characteristic listed, except for sex and gravida, is strongly associated with the type of maternal instruction that was received in this population.

**Table 2:** Descriptive/Laboratory data and associations with maternal jaundice instruction.

Jaundice Instruction	Antenatal and Postpartum	Postpartum Only	None
*N*	337	95	215
Age at Admission, days	4 [3, 6]	7 [4.5, 12.5]	5 [4, 8] *
Admission Weight, grams	2945.0 (666.4)	2902.15 (611.21)	2800.0 (581.7) **
Sex			***
Male	203 (60.2)	60 (63.2)	133 (61.9)
Female	129 (38.3)	35 (36.8)	78 (36.3)
Missing data	5 (1.5)	0 (0.0)	4 (1.9)
Mother’s Age	29.08 (5.99)	26.04 (5.85)	28.00 (6.26) *
Maternal Education			*
Both Pre and Post	337 (100.0)	0 (0.0)	0 (0.0)
Post only	0 (0.0)	95 (100.0)	0 (0.0)
No Education	0 (0.0)	0 (0.0)	215 (100.0)
Number Prenatal Visits			*
0	0 (0.0)	2 (2.1)	22 (10.2)
1 to 3	35 (10.4)	27 (28.4)	39 (18.1)
4 to 5	140 (41.5)	53 (55.8)	82 (38.1)
6 or more	138 (40.9)	12 (12.6)	61 (28.4)
Missing data	24 (7.1)	1 (1.1)	11 (5.1)
Birth Site			*
Hospital	263 (78.0)	35 (36.8)	106 (49.3)
Clinic	31 (9.2)	22 (23.2)	43 (20.0)
Home/Other	42 (12.5)	38 (40.0)	66 (30.7)
Birth Attendant			*
Physician/Midwife	283 (84.0)	57 (60.0)	109 (50.7)
CHEW	48 (14.2)	33 (34.7)	60 (27.9)
TBA/Other	5 (1.5)	4 (4.2)	46 (21.4)
ABE Status			*
No ABE	333 (98.8)	79 (83.2)	152 (70.7)
Mild ABE	3 (0.9)	4 (4.2)	21 (9.8)
Moderate/Severe ABE	1 (0.3)	9 (9.5)	34 (15.8)
Death	0 (0.0)	3 (3.2)	8 (3.7)
Center			*
Jos	173 (51.3)	3 (3.2)	34 (15.8)
Kano	106 (31.5)	79 (83.2)	80 (37.2)
Lagos	19 (5.6)	11 (11.6)	71 (33.0)
Zaria	39 (11.6)	2 (2.1)	30 (14.0)
Laboratory Data			
Admission TSB mg/dL	11.50 [8.45, 15.80]	13.20 [8.6, 18.9]	17.70 [12.55, 25.0] *
Potential Hemolysis			*
No incompatibility	213 (63.2)	59 (62.1)	88 (40.9)
ABO incompatibility	58 (17.2)	20 (21.1)	47 (21.9)
Rhesus incompatibility	12 (3.6)	3 (3.2)	11 (5.1)
Unexplained Hct ≤ 35	3 (0.9)	0 (0.0)	19 (8.8)
Suspected sepsis	0 (0.0)	3 (3.2)	7 (3.3)
Missing Data	50 (14.8)	10 (10.5)	42 (19.5)

*P* values: *<0.001, **0.035, *** 0.743, Legend same as Table 1

### Unadjusted association of maternal instruction with ABE

Maternal instruction and frequency of ABE are strongly associated (chi-squared test, *p* < 0.001) as shown in Table 2. There is a marked reduction of ABE if mothers received combined antenatal with postnatal reinforcement compared with no instruction, and a lesser reduction in ABE if instruction is provided only after birth, the current standard of care. The association remains strong when neonates with mild ABE (bilirubin induced neurological dysfunction (BIND) scores 1–3) were excluded leaving 55 patients with moderate to severe ABE (*p* < 0.001, not shown). The association also remains strong if only patients with four or more clinic visits are included (*p* < 0.001, not shown).

The strong association between maternal instruction and ABE, however, could be due in part to confounding because of the strong associations among ABE, maternal instruction, and socio-economic associated risks (Tables 1, 2), particularly birth site, birth attendant and receipt of antenatal care. These potential confounding factors were examined more closely.

### Impact of antenatal care

The number of prenatal visits is strongly associated with frequency of ABE (Table 1, *p* < 0.001). The number of prenatal visits is also strongly associated with type of maternal instruction (Table 2, *p* < 0.001). Mothers who had no prenatal visits were more likely to have received no instruction, while those who had four or more visits were more likely to have received both antenatal and postpartum instruction. This is expected since the antenatal instruction was delivered at prenatal visits.

### Impact of birth site

The frequency of ABE varies strongly with type of birth site. Newborns admitted for jaundice following out-of-hospital birth had a higher percentage of ABE than infants born in a hospital (Table 1). The association is strong whether the comparison is done using three levels, hospital, clinic and home/other (Table 1, *p* < 0.001), or using two levels, hospital vs. out-of-hospital (*p* < 0.001, not shown). Birth site is also strongly associated with type of maternal instruction (Table 2, *p* < 0.001). Mothers who gave birth in a hospital were much more likely to have had both antenatal and postpartum instruction, while those who gave birth at home were more likely to have had no instruction.

### Impact of birth attendant

The frequency of ABE varies strongly with type of birth attendant. Births of neonates with ABE were less likely to have been attended by a physician or midwife, and more likely to have been attended by a community health worker, traditional birth attendant (TBA) or family member, as shown in Table 1 (*p* < 0.001).

The type of birth attendant is also strongly associated with type of maternal education (Table 2*p* < 0.001). Mothers whose birth was attended by a physician or midwife were more likely to have had both ante- and postpartum instruction, while those attended by a CHEW, TBA, or family member were more likely to have had no instruction.

### Adjusted association of maternal instruction with ABE

Because both frequency of ABE and maternal jaundice instruction type are strongly associated with number of prenatal visits, birth site, and birth attendant, as shown above, the potential for confounding is high. Multiple logistic regression models were therefore used to estimate the effect of maternal instruction type on the odds of ABE after controlling for number of prenatal visits, birth site, birth attendant, and center.

In the adjusted model, type of jaundice instruction was strongly associated with ABE (Type III ANOVA test, *p* < 0.001). Number of prenatal visits (Type III ANOVA test, *p* = 0.001), birth site (Type III ANOVA test, *p* = 0.028), and birth attendant (Type III ANOVA test, *p* = 0.029) were also associated with ABE. Study site, which was strongly associated with ABE before adjustment (Table 1, chi-square test, *p* < 0.001), was no longer associated with ABE after adjusting for the other factors (Type III ANOVA test, *p* = 0.236).

The results for the type of jaundice instruction for both unadjusted and adjusted regression models are shown in Table 3. Looking at the effect of no jaundice instruction the unadjusted odds of having ABE at admission are 2.046 times higher in neonates whose mothers received no jaundice instruction, compared to those whose mothers received the standard postpartum instruction (*p* = 0.022). After controlling for number of prenatal visits, birth site, birth attendant, and center, however, the adjusted odds of ABE for no instruction are only 1.201 times higher (20.1% larger) in neonates whose mothers received no jaundice instruction, compared to those whose mothers received the standard postpartum instruction, and are no longer statistically significant (*p* = 0.637). The apparent strong effect of no instruction on ABE before adjustment may be due to the strong interrelationships among no instruction, few prenatal visits, out-of-hospital birth, and not being attended by a physician or midwife.

**Table 3:** Odds ratios for acute bilirubin encephalopathy (ABE) in admitted neonates as a function of the type of jaundice instruction the mother received.

	Unadjusted Model		Adjusted Model^a^	
	Odds Ratio (95% CI)	*P*-value	Odds Ratio (95% CI)	*P*-value
Postpartum Instruction Only	Reference		reference	
Antenatal and Postpartum Instruction	0.059 (0.017, 0.167)	<0.001	0.104 (0.027, 0.325)	<0.001
No Instruction	2.046 (1.132, 3.878)	0.022	1.201 (0.565, 2.622)	0.637

^a^Adjusted for number of prenatal visits, birth site, birth attendant, and center.

In contrast, the effect of adding antenatal instruction to postpartum instruction is beneficial and remains strong even after adjustment. The unadjusted odds of having ABE at admission are 0.059 times as large (94.1% smaller) in infants whose mothers received both antenatal and postpartum instruction, compared to those whose mothers received the standard postpartum instruction only, and this reduction is statistically significant (*p* < 0.001). This association remains strong even after controlling for number of prenatal visits, birth site, birth attendant, and center: the adjusted odds of ABE are 0.104 times as large (89.6% smaller) in infants whose mothers received both antenatal and postpartum instruction, compared to those whose mothers received the standard postpartum instruction only, and this reduction is statistically significant (*p* < 0.001). This result indicates that adding antenatal instruction to postpartum instruction significantly reduces the odds of ABE, compared to the standard postpartum instruction, over and above the effects of number of prenatal visits, birth site, and birth attendant. Regardless of birth attendant type, type of birth site or number of prenatal visits, adding antenatal instruction to postpartum instruction reduces the odds of ABE in admitted infants by about 90% on average.

### Does maternal instruction increase the number of admissions?

TSB levels were below study guidelines for phototherapy (12 mg/dL) in 30.7% of admissions when mothers received any training and 14.4% if mothers did not receive instruction (*p* < 0.001). If the 2022 American Academy of Pediatrics (AAP) phototherapy guidelines^2^ had been followed, 329/647 (50.8%) would have been admitted “unnecessarily” but would have included four cases of ABE. When only those subjects meeting study treatment guidelines were analyzed, ABE occurred in 4/236 (1.7%), 16/63 (25.4%), and 63/184 (34.2%) in combined, postnatal only, and no instruction groups respectively. Although incidences increased in all groups, differences remained very significant (*p* < 0.001) and conclusions did not change.

## Discussion

This observational study identified two significant associations with the incidence of ABE: risky maternal care choices (home delivery, lack of antenatal care, and untrained birthing attendants) increased the risk for ABE while exposure of mothers to combined antenatal/postpartum instruction decreased the incidence of ABE and mitigated these risks. Although the numbers were small, postnatal instruction did not significantly improve outcome over no instruction so long as mothers had good antenatal care. Large differences in the incidence of ABE were observed in participating centers. Massey Children’s Hospital, a referral hospital in Lagos serving a city with a population of more than 15 million, has no obstetrical service and offered maternal training only in local clinics. All other centers were university based general hospitals serving varying sized populations and health care networks. Differences in ABE rates among centers were not significant when adjusted for behavioral risk factors and rates of maternal instruction.

Content, conduct, and timing (what, how, when) appear to be critical for optimizing the impact of maternal instruction. Because ABE is a present danger in Nigeria, content of teaching materials and discussion emphasized how to identify jaundice, the need to seek evaluation immediately, and consequences of delay (ABE/KSD). Although mothers were taught about causes of jaundice and avoidance of hemolytic triggers (anticipating possible G6PDd), hematologic considerations played little role in most jaundice management decisions. Hematocrits and ABO and Rh incompatible blood types were determined but without direct antibody test confirmation and G6PD activities were not assayed. In cases of suspected G6PDd and Rh hemolytic disease further evaluation and parent counseling was performed in follow up clinics. Most significantly, compared with traditional postpartum instruction alone, the addition of interactive group discussion in antenatal clinics was associated with a significant decrease in ABE among hospital admissions.

Promoting parent instruction about risks and nature of neonatal jaundice is not new. We reviewed several published surveys and reviews revealing deficiencies in both parent and provider knowledge, attitudes and actions regarding neonatal jaundice.^26–32,36–41^ Despite repeated assertions of the need to improve education of mothers and health workers and some publications documenting a beneficial relationship between mothers’ knowledge and timely admission for treatment,^8,41^ we did not identify any systematic investigation relating ante- or post-natal teaching programs to the incidence of hyperbilirubinemia or ABE. In Nigeria, the source of information about jaundice varies but often is from community health workers.^8,41^ Worldwide, postpartum instruction in hospitals varies both in existence and substance, and antenatal instruction about jaundice, demonstrated to be highly effective in our studied population, is still rare. One randomized study in China found a positive relationship of antenatal training and knowledge retention a month following birth but no information on its effect on ABE was presented.^42^

Most published guidelines for managing neonatal jaundice focus on provider management with limited attention to parent involvement. That is beginning to change; the AAP 2022 Clinical Practice Guideline Revision now states, “Before discharge, all families should receive written and verbal education about neonatal jaundice,” and the new AAP parent handout reveals the possibility that elevated bilirubin could cause brain damage. The potential advantage of antenatal education is not addressed.^2^

The slightly older National Institute for Health and Care Excellence (NICE) guidelines (2016) recommend parents be provided information “that is tailored to their needs and expressed concerns…Care should be taken to avoid causing unnecessary anxiety … [and] include [the fact that] neonatal jaundice is common and is usually transient and harmless”.^43^ They advise that parents and caregivers be taught how to check for jaundice and what to do when jaundice is suspected but lack specific parental instructions about the risk of ABE or brain damage.

The World Health Organization (WHO) 2022 recommendations on maternal and newborn care advise “written/digital education booklets, pictorials for semi-literate populations and job aids should be available…. Parents and family should be encouraged to seek health care early if they identify danger signs between postnatal care visits.” Listed danger signs relevant to jaundice management include not feeding well; no spontaneous movement; convulsions; fever; any jaundice in first 24 h after birth, and yellow palms and soles at any age.^44^ TSB is frequently greater than 15–20 m/dL when hands and feet are jaundiced.^45^ The listed danger signs reflect both early and advanced, often irreversible, ABE as defined by BIND score assessment.^33,34^

The most recent 2021 Nigerian National Guidelines for Comprehensive Newborn Care is the first guideline recommending “Mothers should be provided with verbal and written information during antenatal care and at postnatal discharge on the dangers of neonatal jaundice and need to urgently bring baby for assessment in health facility”.^34^

A potential downside to maternal empowerment, confirmed in this study, is increased care-seeking resulting in unnecessary admissions. When mothers received combined instruction, 31% were treated with bilirubin levels below the modest phototherapy indicator of 12 mg/dL. But, had we applied 2022 AAP guidelines, rejection of admission would have doubled and included four patients with ABE, reinforcing the view that LMIC guidelines must be tailored to risks and medical care barriers documented in each region.^2,46^ While educating mothers to seek care anytime their neonate has evidence of jaundice is crucial, the resulting excess admissions highlights the need to provide community healthcare workers the skill to screen jaundiced neonates locally. Screening tools such as icterometers, transcutaneous monitors, and smartphone-based point-of-care devises could improve selecting patients who require further evaluation and reduce the burden on overtaxed hospital systems. Kramer scores are inaccurate in detecting significant jaundice, especially in deeply pigmented newborns.^45^

### Limitations

This is not a randomized, controlled prospective study and is limited to neonates admitted at participating centers for treatment of jaundice. Although the study coincided with and was designed to evaluate the introduction of antenatal training, comparison group selection was defined retrospectively using opportunistic controls as noted previously.^23^ We identified contributing risk choices and behaviors that are likely social-economic markers but did not document other SE criteria such as education level, income, or literacy. We did not obtain population data—how many received instructions without follow up assessment; how many were not offered antenatal training versus chose not to participate; how many parents did not seek care for their neonate with mild ABE. While mild ABE might not always initiate care seeking, it is less likely that neonates with moderate to severe ABE or death would escape medical attention.

Notwithstanding these reservations, the presented evidence supports what some have termed common sense—that anticipatory guidance in late pregnancy with reinforcement following birth is more effective than traditional postpartum instruction in enhancing jaundice care skills and appropriate actions by parents. This likely holds true in HICs as well as LMICs.^22^ Introducing the program as a cluster-randomized study would further verify its impact.

## Conclusions

Antenatal interactive group presentations of information about jaundice and its risks, combined with postpartum reinforcement, was associated with improved parent care seeking (earlier admission) and a lower incidence of severe jaundice/hyperbilirubinemia and ABE in a LMIC where progression to KSD is an all too common and largely preventable tragedy.

We recommend that antenatal clinics incorporate interactive jaundice instruction into their prenatal class curriculum together with postnatal reinforcement, especially in regions where ABE is common. Consensus-driven uniform jaundice instruction modules for healthcare providers and parents should be developed by relevant professional bodies. The expense of implementing this program is small compared to the human and care costs of supporting children with ABE/KSD.

### Supplementary information

SKIN_Members_List

## Data Availability

The datasets (Excel files) analyzed in the current study are available from the corresponding author on request.
