# Supplementary material for: Antenatal jaundice instruction and acute bilirubin encephalopathy in Nigeria
Source: Pediatr Res. 2023 Dec 2;95(5):1301–7. doi: 10.1038/s41390-023-02887-6 (PMC11035125; doi:10.1038/s41390-023-02887-6)
Supplement: Supplementary file 1 — SKIN_Members_List [file 41390_2023_2887_MOESM1_ESM.pdf]

Stop Kernicterus in Nigeria (SKIN) member list:

Angela A Okolo A

William N Ogala

Fidelia Bode-Thomas

Bose O Toma

Christopher S Yilgwan

Akinyemi O Ofakunrin

Udochukwu M Diala

Chris Isichei

Victor Pam

Zuwaira Hassan

Shehu U Abdullahi

Fatima Usman

Binta W Jibir

Idris Y Mohammed

Hadiza Usman

Muhammed Abdusalam

Aisha Kuliya-Gwarzo

Fatima Isiga-Ahmad

Laila Umar

Fatima Abdullahi

Carlos D Coda-Zabetta
